# Patient Experiences with the Transition to Telephone Counseling during the COVID-19 Pandemic

**DOI:** 10.3390/healthcare9060663

**Published:** 2021-06-02

**Authors:** Augustine W. Kang, Mary Walton, Ariel Hoadley, Courtney DelaCuesta, Linda Hurley, Rosemarie Martin

**Affiliations:** 1 Center for Alcohol and Addiction Studies, Brown University School of Public Health, Providence, RI 02912, USA; Courtney_delacuesta@brown.edu (C.D.); rosemarie_martin@brown.edu (R.M.); 2 CODAC Behavioral Healthcare, Cranston, RI 02910, USA; mwalton@codacinc.org (M.W.); lhurley@codacinc.org (L.H.); 3 Department of Social and Behavioral Sciences, Temple University College of Public Health, Philadelphia, PA 19122, USA; ariel_hoadley@brown.edu

**Keywords:** opioid use disorder treatment, telehealth services, qualitative, needs assessment

## Abstract

*Background:* To identify and document the treatment experiences among patients with opioid use disorder (OUD) in the context of the rapid move from in-person to telephone counseling due to the COVID-19 pandemic. *Methods:* Participants (*n* = 237) completed a survey with open-ended questions that included the following domains: (1) satisfaction with telephone counseling, (2) perceived convenience, (3) changes to the therapeutic relationship, (4) perceived impact on substance use recovery, and (5) general feedback. Responses were coded using thematic analysis. Codes were subsequently organized into themes and subthemes (covering 98% of responses). Interrater reliability for coding of participants’ responses ranged from 0.89 to 0.95. *Results:* Overall, patients reported that telephone counseling improved the therapeutic experience. Specifically, 74% of respondents were coded as providing responses consistently indicating “positive valency”. “Positive valency” responses include: (1) feeling supported, (2) greater comfort and privacy, (3) increased access to counselors, and (4) resolved transportation barriers. Conversely, “negative valency” responses include: (1) impersonal experience and (2) reduced privacy. *Conclusions*: Telephone counseling presents its own set of challenges that should be investigated further to improve the quality of care and long-term patient outcomes.

## 1. Introduction

The ongoing opioid epidemic and the COVID-19 pandemic constitute a syndemic [1]. More than 40 states in the United States have reported increases in opioid-related mortality in the first six months of the pandemic, which has become more complicated and deadly as the pandemic persists [2].

Medications for opioid use disorder (MOUD) are among the most systematically governed treatment approaches in the United States [3]. Although MOUD is the evidence-based standard of care for OUD, access is limited primarily due to the strict federal and state regulations mandating in-person medical and clinical encounters to initiate and maintain MOUD. However, the COVID-19 public health emergency led to an immediate cascade in relaxing laws, regulations, and policies to enable ongoing treatment by reducing financial and administrative obstacles and expanding the role of telemedicine, to name a few [4]. These changes resulted in shifts in the access and delivery of MOUD, providing an opportunity to improve treatment and thus reduce opioid-related morbidity and mortality in a time of national crisis.

Rates of telehealth services provisions for substance use disorder (SUD) had been generally low before COVID-19 even though telehealth services increase patient access, adherence, and retention in treatment while yielding equivalent outcomes to in-person care [5,6]. For OUD treatment specifically, some evidence indicates similar rates of counseling attendance, drug-positive urinalysis results, and retention in treatment between telehealth versus in-person-based provision of services [7,8].

As CODAC Behavioral Healthcare, Inc., the largest outpatient opioid treatment organization in the state of Rhode Island (USA), transitioned from in-person encounters to a virtual telephonic platform, it was unclear how the therapeutic relationship between patients and counselors would change as a result. Positive therapeutic relationships (or alliances) are important for treatment engagement as it indicates (1) high-quality interactions between patients and their counselors, (2) personal bonds between patients and their counselors, and (3) a collaborative relationship of task and goal development for the patients’ substance use recovery journey [9]. Hence, CODAC and Brown University partnered to conduct patient satisfaction surveys to explore patients’ perspectives on telephone counseling during the pandemic. The aim of this paper is to qualitatively examine patient responses to open-ended survey questions to gain insight into how telephone counseling may have impacted the patients’ treatment experience.

## 2. Materials and Methods

*Study Design*. The present study examines data from a larger quality improvement project (at CODAC) to assess patient and counselor experiences with telephone counseling in the context of COVID-19 risk mitigation. Cross-sectional survey data was used to understand the experiences of patients, counselors, and prescribers who had participated in telephone counseling sessions and/or provided services to patients across seven opioid treatment program (OTP) clinics (under the ambit of CODAC) located across Rhode Island during the COVID-19 pandemic. Data from administrative records included insurance status, age, gender, and race/ethnicity. Counseling services were required at least once per month and could receive one of the three FDA-approved medications for opioid use disorder: methadone, buprenorphine, or extended-release naltrexone. All data were de-identified. The CODAC research oversight committee reviewed and approved the project.

*Participants.* From 3 July to 8 November 2020, prospective participants were invited to complete the survey via their CODAC-based counselors during routine telephone counseling sessions or via OTP staff in-person at the clinic. Patients who provided verbal consent to counselors received an invitation via text message to participate in a web-based, patient satisfaction survey. Patients approached in-person at the clinics completed paper surveys. All patients who completed the survey were entered into raffles at each treatment site for a $25 gift card.

Approximately 16% of all CODAC patients who had at least one telephone counseling session at a clinic from 16 March to 8 November 2020 and who received in-person counseling prior to the COVID-19 mitigations completed the survey. The survey included five open-ended questions that queried the following domains respectively: (1) satisfaction, (2) convenience, (3) relationship with their counselor, (4) substance use and recovery, and (5) general feedback (Table 1).

*Qualitative Analysis Approach.* Open-ended responses to the five questions described above were coded by two independent raters following the principles of inductive thematic analysis [10]. Specifically, both raters read all responses, assigned preliminary codes to the texts, and then discussed emergent codes and themes collectively with the study authors. A codebook containing two major themes and four subthemes was developed via an iterative coding process (i.e., assigning and re-assigning the names of codes if necessary, taking into account the context of emerging evidence as the qualitative coding process proceeded) covering all five open-ended questions. Two other raters then subsequently re-applied the codebook to the open-ended responses into codes and themes. The final codebook covered 98% of patients’ responses. Interrater reliability for coding of patient responses ranged from 0.89 to 0.95.

## 3. Results

Participant characteristics are presented in Table 2.

**Figure 1:**
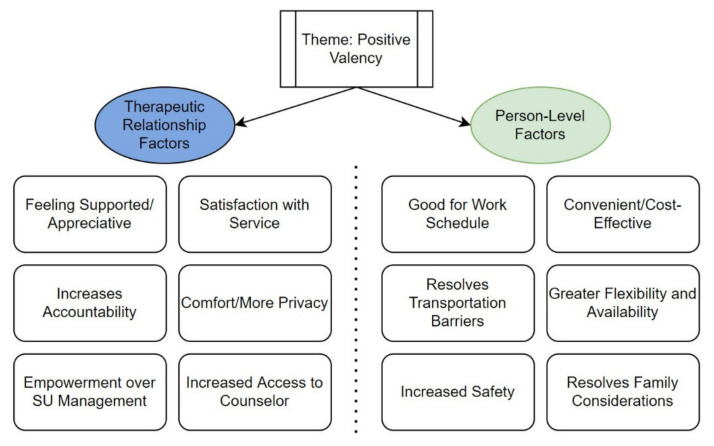
Overview of subthemes and codes—positive valency.

**Figure 2:**
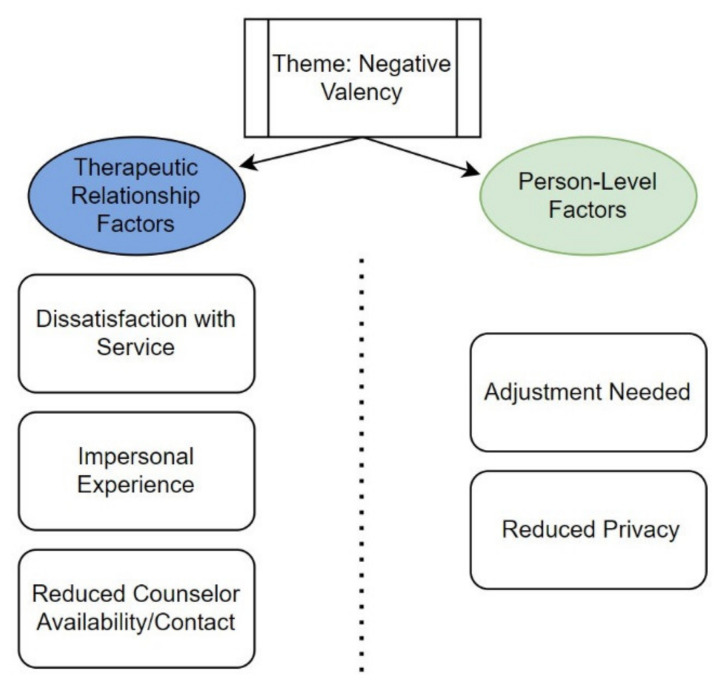
Overview of subthemes and codes—negative valency.

Figure 1 and Figure 2 summarize the results of the analysis. The codes/subthemes were grouped into two overall themes: “positive valency” and “negative valency”. Participants were grouped into “positive valency” and “negative valency” if their responses across the five open-ended questions were consistently coded as positive or negative, respectively. To create a more parsimonious narrative, we grouped “mixed” valency responses (i.e., there were both positive and negative valence responses across the five questions at the participant level) with “negative” valency. “Neutral” participants (*n* = 10) were defined as providing answers that reflected indifference (e.g., “the same”) and were excluded from further discussion. (Table 2).

Within each valency theme, two similar subthemes were observed: (1) therapeutic relationship factors (defined as factors impacting the relationship and/or process of interaction between counselor and patient) and (2) person-level factors (defined as factors operating at the individual level that impact the counseling experience).

### 3.1. Positive Valency—Therapeutic Relationship Factors

Participants reported that they felt supported by their counselors and were appreciative of their efforts during the transition into telephone counseling. One participant wrote, “*My counselor goes above and beyond to make sure I have everything I need during this troubling time”* (P03, or Participant 03). Participants also described how despite the transition to telephone counseling and the lack of in-person contact they remained satisfied with service. For example, one participant wrote, “*I love my counselor. I can be completely honest with her on the phone or in-person*” (P02). Some participants also explained that telephone counseling provided more comfort and privacy relative to in-person settings. For example, “*Feel more comfortable because for me I am shy so talking on the phone feels more comfortable”* (P01). Increased access to one’s counselor (i.e., a greater sense of connectedness) was also reported, such as, “*We talk more on the phone than we do in person”* (P10) and “*…but as I said before I think it’s easier to talk to her more over the phone if need be”* (P05). In addition, some participants also report increased accountability to their counselor (and perhaps, by extension, to their recovery journey) due to frequency of counseling, “*We talk more. It used to be once a month…now I get to talk to [Redacted, name of counselor] once a week. This way nothing gets missed and nothing get[s] unmentioned”* (P06) and feeling empowered over their substance use management (*“I feel now in control of my recovery by not having the feeling that I need to be somewhere at a certain time”* (P17)).

### 3.2. Positive Valency—Person-Level Factors

**Figure 3:**
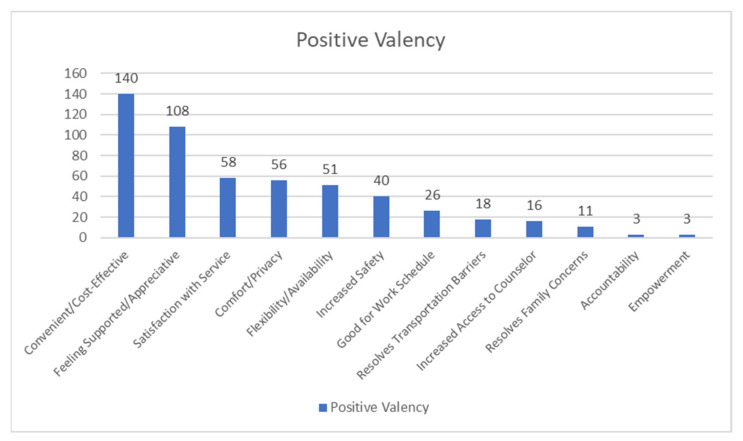
Frequencies of codes—positive valency.

Many participants described that telephone counseling made the experience of receiving treatment more convenient compared to in-person counseling. For example, one participant explained that telephone counseling was “…*quick and easy, no lines to wait in*” (P18), indicating that telephone counseling may be more time-effective for some. Furthermore, participants also reported that telephone counseling made it easier to manage one’s work schedule. For example, one participant mentioned, “…*it’s the easiest way instead of having to take time out of work I can just step away for a phone call”* (P09). Participants who likely do not have reliable personal transportation methods also said that telephone counseling resolved previously experienced transportation barriers, such as one who explained that it was, “…*more convenient because I don’t need to drive or get a ride”* (P04). Telephone counseling also resolved time-related family considerations (e.g., “*Don’t have to drag my kids out”* (P11)). In addition, participants also mentioned that telephone counseling allowed for more flexibility compared to in-person counseling (e.g., “*It fits my schedule better and doesn’t make counseling and dosing related”* (P12)). Lastly, participants recognized that, amidst the pandemic, switching to telephone counseling provided a sense of safety (e.g., “…*keeps me from getting COVID-19*” (P13)). Figure 3 summarizes the frequencies of codes for the theme of positive valency.

### 3.3. Negative Valency—Therapeutic Relationship Factors

Some participants were generally dissatisfied with telephone counseling (e.g., “*Every time I ask my counselor for help they took a long time or forgot”* (P16). Many participants explained that telephone counseling felt more impersonal compared to in-person counseling. For example, one participant described that “*Most issues can be handled by telephone, but obviously sometimes physical presence is required…there is something lost between the counselor and client. Certainly [even more so] for new clients who have not yet built a rapport with their counselors*” (P15). Some participants also reported reduced counselor contact after switching to telephone counseling (e.g., “*Less contact”* (P14) and *“Don’t get many calls”* (P07)).

### 3.4. Negative Valency—Person-Level Factors

**Figure 4:**
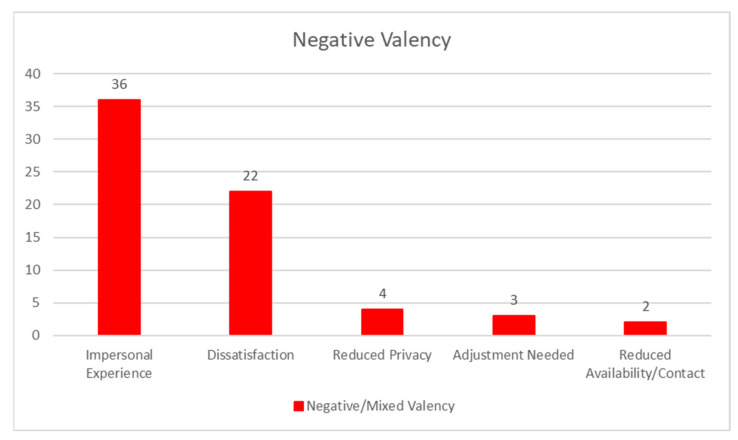
Frequencies of codes—negative valency.

Participants also explained that they may not receive adequate privacy at home for counseling. In addition, some participants also mentioned that an adjustment to telephone counseling was necessary. For example, “*Initially, I was a bit hesitant because I wasn’t home alone. However, once I worked out at-home privacy issues, I felt more confident talking and working things out*” (P08). Figure 4 summarizes the frequencies of codes for the theme of negative valency.

## 4. Discussion

Results suggest that telephone counseling for MOUD may facilitate the therapeutic experience and treatment engagement among patients. However, our analysis also identified that telephone counseling presents its own set of challenges that may undermine treatment experiences and should be investigated further to improve the quality of care and long-term patient outcomes among the MOUD patient population.

Our findings suggest telephone counseling fostered a sense of convenience, support, and comfort (in terms of discussing one’s substance use recovery), which is consistent with previous research examining telephone counseling approaches for SUDs [11]. These factors may contribute to an improved therapeutic alliance and increase the likelihood of long-term treatment engagement [12]. In the context of MOUD provision, these factors may be beneficial in improving treatment across patient populations [13,14]. Considering that individuals with SUDs experience a gap between treatment need and utilization [15], telephone counseling for MOUD could be a viable way to increase treatment access and engagement to help bridge this gap.

Future efforts to integrate various telehealth approaches in MOUD treatment provision should further explain and proactively mitigate negative patient experiences and potential barriers to virtual treatment engagement. Our results identified perceptions of an impersonal experience for some; impersonal experiences have been reported to predict reduced treatment engagement and a weaker therapeutic alliance between patient and provider [16]. While an impersonal experience has consistently been reported in the telemedicine experience, to our knowledge no papers have reported and/or explored how perceptions of an impersonal experience with telehealth counseling for MOUD treatment may impact treatment engagement and outcomes.

Some limitations of our study should be noted. The cross-sectional study design restricted our ability to examine changes in the patients’ perspectives toward telephone counseling. For example, it is unclear if fatigue with the pandemic and the extended engagement of telephone counseling will adversely affect patients’ perspectives of telephone counseling, or if perspectives toward telephone counseling will change post-pandemic. In addition, the survey required patients to recall their counseling experiences pre-pandemic and contrast them with their current telephone counseling experience, which may have introduced some recall bias. Patients who did not complete any telephone counseling sessions were not eligible to participate in the study, which may have limited the scope of our data. Furthermore, our study population was primarily White and was limited to the geographical region of Rhode Island (USA), limiting the generalizability of our findings. Finally, we did not include measures of addiction severity and how it may impact therapeutic alliance in the telephone counseling context. Regardless, our findings fill a key gap in the literature in illustrating the perspectives among patients about the transition to telephone counseling.

## 5. Conclusions

Most patients in our study reported a positive experience with using telephone counseling for OUD treatment. In the post-pandemic setting, adopting a hybrid in-person/telehealth approach may be one way to assuage concerns regarding an impersonal experience. The expansion of the traditional system of in-person care delivery models into telephone counseling due to the pandemic holds significant promise for improving accessibility to and management of MOUD treatment among the patient population. Future research should adopt current in-person MOUD provision models and tailor evidence-based approaches to the unique nuances of the telehealth (or a hybrid telehealth/in-person) service approach.

## Figures and Tables

**Table 1:** Open-ended questions.

Domain	Question
Satisfaction	How satisfied are you currently with your telephone counseling sessions? Please tell us why you feel this satisfaction level.
Convenience	How convenient is telephone counseling for you compared to being in the office face-to-face? Please tell us why it is more, the same, or less convenient.
Relationship with counselor	Please describe how your relationship with your counselor may have changed using telephone counseling.
Substance use and recovery	If you would like to explain how counseling has or has not helped in your substance use or recovery, please do so.
General feedback	Is there anything else you would like to tell us about your counseling experiences during the pandemic?

**Table 2:** Demographics and valency characteristics (*n* = 237).

Variable	*M* ± *SD*/*n* (%)	Valency		*p*
		Positive	Mixed/Negative ^ƚ^	
Valency of responses		184 (78)	53 (22)	
Age	41.7 ± 10.2 years	41.3 ± 10.1	41.7 ± 11.8	0.813 ^a^
Insurance status *				
Medicaid/State-based	74%	NA	NA	
Medicare	6%			
Commercial	14%			
Self-pay	6%			
Gender				
Male	105 (52)	83 (52)	22 (50)	0.798 ^b^
Female	98 (48)	76 (48)	22 (50)	
Race/Ethnicity				
White, non-Hispanic	133 (81)	100 (79)	33 (90)	0.562 ^b^
Black, non-Hispanic	6 (4)	5 (4)	1 (3)	
Hispanic	16 (10)	15 (12)	1 (3)	
Unknown	9 (5)	7 (5)	2 (4)	

Notes: Missing data for gender = 34 and for race/ethnicity = 73. ƚ Negative-only participants were *n* = 29 (12% of total responses). * Insurance status was PHI and hence was not matched to participant IDs in this survey. Figures presented are aggregated data for the broader participant population. ^a^ Independent samples *t*-test. ^b^ Chi-square analysis.

## Data Availability

The data presented in this study are available on request from the corresponding author. The data are not publicly available due to privacy and ethical considerations.

## References

[1] Becker S.J., Garner B.R., Hartzler B.J. (2021). Is necessity also the mother of implementation? COVID-19 and the implementation of evidence-based treatments for opioid use disorders. J. Subst. Abuse Treat..

[2] AMA Advocacy Resource Center (2020). Issue Brief: Reports of Increases in Opioid-and Other Drug-Related Overdose and Other Concerns During COVID Pandemic.

[3] Substance Abuse and Mental Health Services Administration (SAMHSA) (2018). Medications for Opioid Use Disorder. Treatment Improvement Protocol (TIP).

[4] Hughto J.M., Peterson L., Perry N.S., Donoyan A., Mimiaga M.J., Nelson K.M., Pantalone D.W.T. (2021). The provision of counseling to patients receiving medications for opioid use disorder: Telehealth innovations and challenges in the age of COVID-19. J. Subst. Abuse Treat..

[5] Backhaus A., Agha Z., Maglione M.L., Repp A., Ross B., Zuest D., Rice-Thorp N.M., Lohr J., Thorp S.R. (2012). Videoconferencing psychotherapy: A systematic review. Psychol. Serv..

[6] Batastini A.B., Jones A.C., Lester M.E., Davis R.M. (2020). Initiation of a multidisciplinary telemental health clinic for rural justice-involved populations: Rationale, recommendations, and lessons learned. J. Community Psychol..

[7] King V.L., Brooner R.K., Peirce J.M., Kolodner K., Kidorf M.S. (2014). A randomized trial of web-based videoconferencing for substance abuse counseling. J. Subst. Abuse Treat..

[8] Eibl J.K., Gauthier G., Pellegrini D., Daiter J., Varenbut M., Hogenbirk J.C., Marsh D.C. (2017). The effectiveness of telemedicine-delivered opioid agonist therapy in a supervised clinical setting. Drug Alcohol Depend..

[9] Digiuseppe R., Linscott J., Jilton R. (1996). Developing the therapeutic alliance in child-adolescent psychotherapy. Appl. Prev. Psychol..

[10] Clarke V., Braun V. (2014). Thematic Analysis. Encyclopedia of Critical Psychology.

[11] Steinkamp J.M., Goldblatt N., Borodovsky J.T., Lavertu A., Kronish I.M., Marsch L.A., Schuman-Olivier Z. (2019). Technological interventions for medication adherence in adult mental health and substance use disorders: A systematic review. JMIR Mental Health.

[12] Seal K.H., Abadjian L., McCamish N., Shi Y., Tarasovsky G., Weingardt K. (2012). A randomized controlled trial of telephone motivational interviewing to enhance mental health treatment engagement in Iraq and Afghanistan veterans. Gen. Hosp. Psychiatry.

[13] Schinke S.P., Fang L., Cole K.C.A., Cohen-Cutler S. (2011). Preventing substance use among Black and Hispanic adolescent girls: Results from a computer-delivered, mother–daughter intervention approach. Subst. Use Misuse.

[14] Ryan-Pettes S.R., Lange L.L., Magnuson K.I. (2019). Mobile phone access and preference for technology-assisted aftercare among low-income caregivers of teens enrolled in outpatient substance use treatment: Questionnaire study. JMIR mHealth uHealth.

[15] Park-Lee E., Lipari R.N., Hedden S.L., Kroutil L.A., Porter J.D. (2017). Receipt of services for substance use and mental health issues among adults: Results from the 2016 National Survey on Drug Use and Health.

[16] Cox A., Lucas G., Marcu A., Piano M., Grosvenor W., Mold F.E., Maguire R., Ream E. (2017). Cancer survivors’ experience with telehealth: A systematic review and thematic synthesis. J. Med. Internet Res..

